# Peptides Derived from Vascular Endothelial Growth Factor B Show Potent Binding to Neuropilin‐1

**DOI:** 10.1002/cbic.202100463

**Published:** 2021-11-03

**Authors:** Filipa Mota, Tamas Yelland, Jennie A. Hutton, Jennifer Parker, Anastasia Patsiarika, A. W. Edith Chan, Andrew O'Leary, Constantina Fotinou, John F. Martin, Ian C. Zachary, Snezana Djordjevic, Paul Frankel, David L. Selwood

**Affiliations:** ^1^ Wolfson Institute for Biomedical Research University College London Gower Street London WC1E 6BT UK; ^2^ The Institute of Structural and Molecular Biology University College London UK; ^3^ Centre for Cardiovascular Biology & Medicine BHF Laboratories at University College London UK; ^4^ Institute of Cardiovascular Science University College London UK

**Keywords:** bicyclic peptides, lipidated peptides, neuropilin, surface plasmon resonance, VEGF-B

## Abstract

Vascular endothelial growth factors (VEGFs) regulate significant pathways in angiogenesis, myocardial and neuronal protection, metabolism, and cancer progression. The VEGF‐B growth factor is involved in cell survival, anti‐apoptotic and antioxidant mechanisms, through binding to VEGF receptor 1 and neuropilin‐1 (NRP1). We employed surface plasmon resonance technology and X‐ray crystallography to analyse the molecular basis of the interaction between VEGF‐B and the b1 domain of NRP1, and developed VEGF‐B C‐terminus derived peptides to be used as chemical tools for studying VEGF‐B ‐ NRP1 related pathways. Peptide lipidation was used as a means to stabilise the peptides. VEGF‐B‐derived peptides containing a C‐terminal arginine show potent binding to NRP1‐b1. Peptide lipidation increased binding residence time and improved plasma stability. A crystal structure of a peptide with NRP1 demonstrated that VEGF‐B peptides bind at the canonical C‐terminal arginine binding site. VEGF‐B C‐terminus imparts higher affinity for NRP1 than the corresponding VEGF‐A_165_ region. This tight binding may impact on the activity and selectivity of the full‐length protein. The VEGF‐B_167_ derived peptides were more effective than VEGF‐A_165_ peptides in blocking functional phosphorylation events. Blockers of VEGF‐B function have potential applications in diabetes and non‐alcoholic fatty liver disease.

## Graphical abstract

**VEGF‐B binds** to the b1 domain of neuropilin‐1. VEGF‐B‐derived peptides show potent binding to neuropilin‐1 (K_D_=0.13 to 9.55 μM) and inhibit binding of VEGF‐A in a cell‐based assay (IC_50_=0.3 to 2.0 μM). Peptide lipidation increased binding and plasma half‐lives.

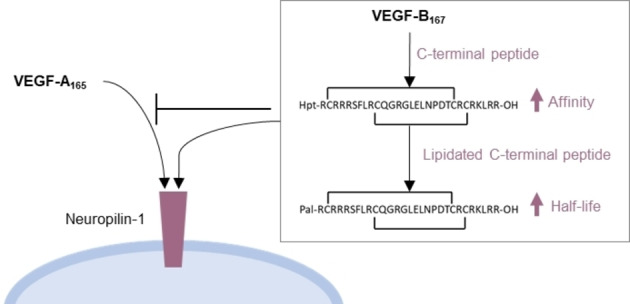

## Introduction

**Figure 1:**
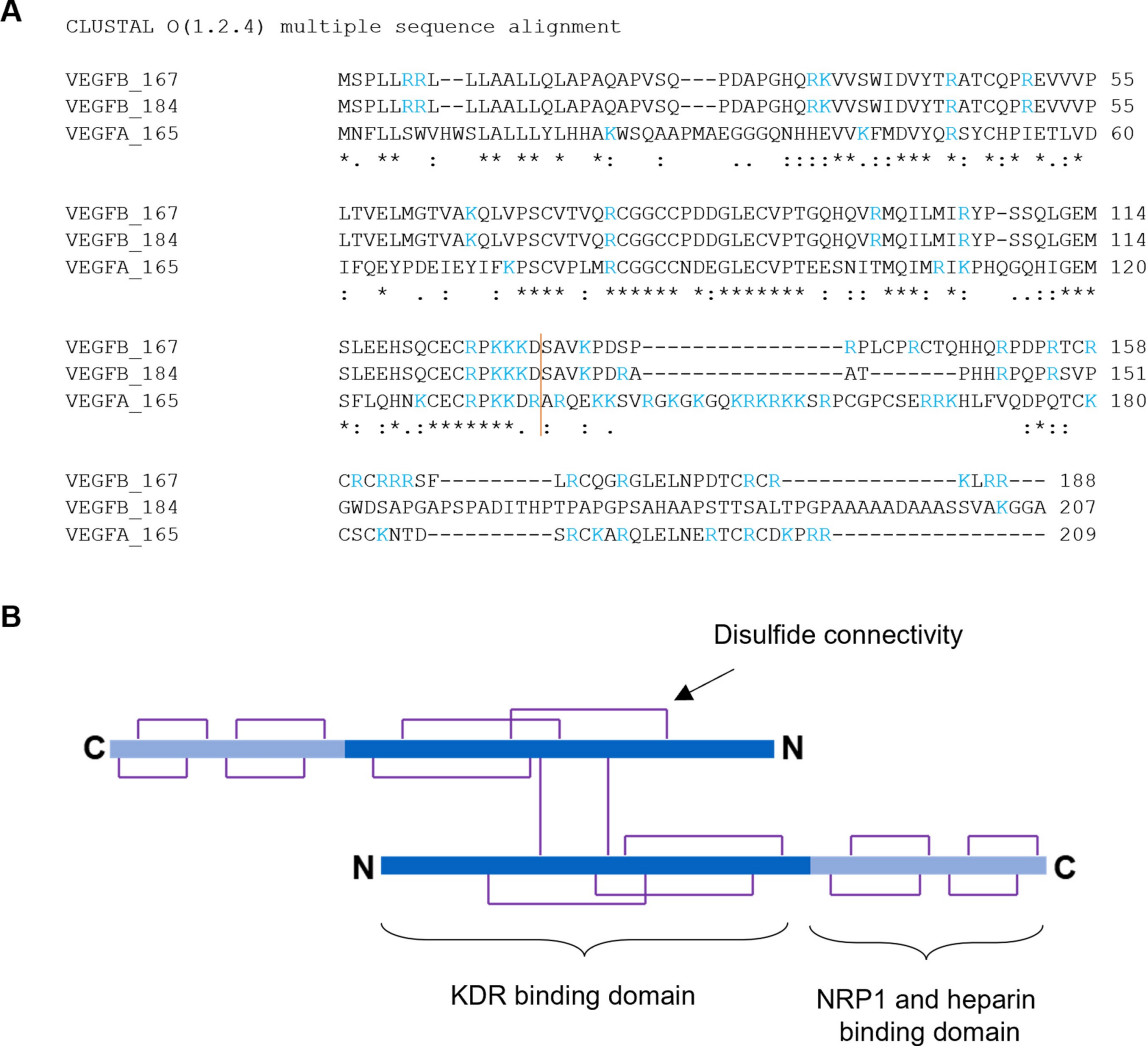
A) alignment of the two major VEGF‐B isoforms with VEGF‐A using the Clustal Omega program (Uniprot https://www.uniprot.org/align) P49765‐2|VEGF‐B_167; SP|P49765‐1|VEGF‐B_186; P15692‐4 VEGF‐A_165_. The orange bar shows the beginning of the heparin binding domain as identified for VEGF‐A_165_ and assumed for VEGF‐B isoforms. DRAATPHHRPQPR region of VEGF‐B_186_ shown in magenta. B) Domain organisation of VEGF isoforms showing the disulfide connectivity, head to tail arrangement and N and C termini.

The vascular endothelial growth factor (VEGF) family, which includes VEFG‐A, VEGF‐B, VEGF‐C, VEGF‐D, and placental growth factor (PlGF), induces cellular responses through binding to the extracellular domain of transmembrane receptors. While the signalling pathways involving VEGF‐A have been extensively studied, the role of VEGF‐B is not fully understood. VEGFs exhibit different binding to VEGF receptors (VEGFRs) and other co‐receptors and are therefore considered to act through distinct mechanisms and have distinct biological and pathophysiological functions. VEGF‐A binds both VEGFR1 and VEGFR2 and its role is mainly associated with angiogenesis. VEGF‐C and VEGF‐D bind to VEGFR2 and VEGFR3 and they are mainly involved in lymphangiogenesis. VEGF‐B and PlGF are thought to bind to VEGFR1 alone.^[1]^ VEGFs can also bind neuropilin‐1 (NRP1) and neuropilin‐2 (NRP2) and NRPs play important roles in the biological functions of VEGFs. VEGF‐B has two isoforms, which are similar in sequence and domain structure (Figure 1A and 1B). VEGF‐B_167_ contains a heparin binding domain making it capable of anchoring to the cell surface, and VEGF‐B_186_, which is soluble.^[2]^ The VEGF‐B_186_ isoform is proteolytically processed to a protein which contains a C‐terminal arginine and this allows increased NRP1 binding.^[3]^

VEGF‐B is dispensable for the formation and growth of blood vessels,^[4]^ but several studies indicate it acts as a cytoprotective agent in the myocardium. Both *in vitro* and *in vivo* models of ischemia‐reperfusion injury have shown that VEGF‐B is able to protect cardiomyocytes by activating Akt and regulating signal transducers involved in apoptosis and autophagy.^[5]^ Additionally, it was found that Doxorubicin‐induced cardiotoxicity is associated with downregulation of VEGF‐B.^[6]^

In cancer, VEGF‐B promotes tumour progression and metastasis.[^7^, ^8^, ^9^] A VEGF‐B α1 helix derivative was able to interact with both VEGFR1 and VEGFR2 implicating the region‘s potential role in angiogenesis and tumour growth.^[10]^ Recently, VEGF‐B has been proposed as a possible biomarker in breast cancer diagnosis.^[11]^

It has recently been shown that VEGF‐B is a potent regulator of antioxidant pathways, which may account for its role in cell survival.^[12]^ Studies performed in preclinical animals models of neurodegeneration have also shown that VEGF‐B has neuroprotective roles[^13^, ^14^] and VEGF‐B derived peptides which bind VEGFR1 may exert a similar effect.^[15]^ VEGF‐B is also known for its regulatory role in metabolism and possible involvement in diabetes, albeit through mechanisms not yet fully understood.[^16^, ^17^] In non‐alcoholic fatty liver disease (NAFLD) VEGF‐B shows a correlation with blood pressure and renal dysfunction and has been proposed as a biomarker of disease.^[18]^ It has also been suggested that VEGF‐B inhibition could have a protective role against diabetic kidney disease and in the treatment of diabetic retinopathy.[^19^, ^20^]

Makinen *et al*. found that VEGF‐B is able to bind to NRP1 and that an excess of VEGF‐A_165_ is able to inhibit this interaction. The binding of VEGF‐B_167_ is thought to be mediated by the heparin binding domain, whereas the binding of VEGF‐B_186_ to NRP1 is regulated by exposure of a short COOH‐terminal proline‐ rich peptide upon its proteolytic processing.^[21]^ A study by Jenssen *et al*. has shown that silencing either VEGF‐B or NRP1 in zebrafish embryos results in virtually the same lethal phenotype characterized by impaired brain development and vasculature, indicating an important role for NRP1 in mediating physiological functions of VEGF‐B. Interestingly, the lethal phenotype was not observed in mice, implicating that different species may have different responses to VEGF‐B/NRP1 signalling.^[22]^

NRP1 is known to act as a co‐receptor for VEGF‐A and Semaphorins. However, the biological roles of the VEGF‐B ‐ NRP1 interaction remain unknown. Inhibitors of these interactions could help elucidate the importance of VEGF‐B/NRP1 signalling in cell homeostasis and may prove useful as treatments in diseases such as cancer and diabetes. We undertook a study to examine the interaction of VEGF‐B with NRP1 using VEGF‐B derived peptides. Since NRP1 possesses a well‐defined arginine‐binding site, we designed these peptides to incorporate the VEGF‐B_167_ C‐terminal arginine. We had previously followed a similar approach to obtain VEGF‐A ‐ derived peptides with potent binding to NRP1.^[23]^ However, further development revealed poor plasma stability. To overcome this, we further modified our VEGF‐B and VEGF‐A ‐ derived peptides with the addition of palmitoyl side chains. Since peptide lipidation can be used to modulate pharmacokinetics and pharmacodynamics, we hypothesised this strategy would stabilize the peptides and slowdown the binding off‐rates, a feature which we expect would facilitate potential translation of these chemical tools. This approach has been successfully employed in diabetes and obesity treatments.[^24^, ^25^] We used surface plasmon resonance (SPR) technology to study the binding interaction between VEGF‐B and NRP1 and to measure the direct binding affinity and kinetics between the novel peptides and NRP1. Immunoblotting was used to assess downstream effects of selected peptides in VEGF‐A ‐ induced phosphorylation, and inhibition of VEGF‐A binding to NRP1 was also measured in HUVEC cells.

## Results and Discussion

### Binding of NRP1‐b1 to immobilised VEGF‐B_167_

**Figure 2:**
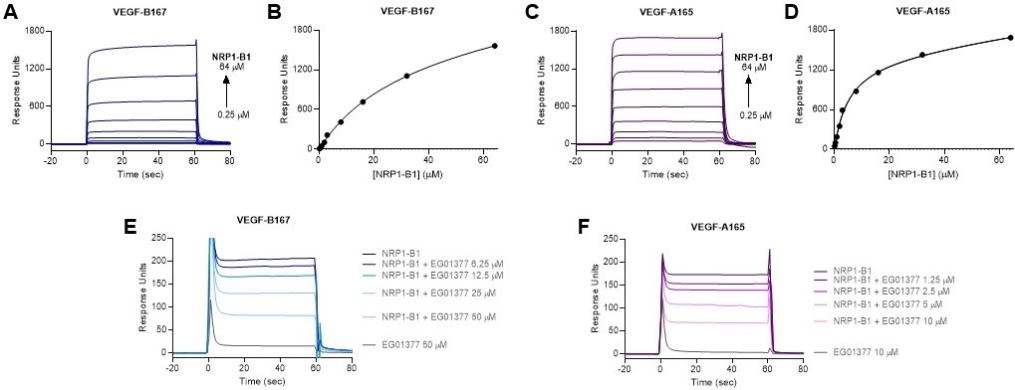
NRP1‐b1 binds to immobilized VEGF‐B_167_ and VEGF‐A_165_, competition with a NRP1 binding peptide. A) SPR sensorgram of NRP1‐b1 (0.25 to 64 μM) binding to VEGF‐B_167_. B) Sensorgram‐derived dose‐response curve of NRP1‐b1 (0.25 to 64 μM) binding to VEGF‐B_167_. C) SPR sensorgram of NRP1‐b1 (0.25 to 64 μM) binding to VEGF‐A_165_. D) Sensorgram‐derived dose‐response curve of NRP1‐b1 (0.25 to 64 μM) binding to VEGF‐A_167_. E) Sensorgrams showing dose‐response inhibition of NRP1‐b1 (5 μM) to VEGF‐B_167_ by EG01377 (6.25 to 50 μM). F) Sensorgrams showing dose‐response inhibition of NRP1‐b1 (5 μM) to VEGF‐A_167_ by EG01377 (1.25 to 10 μM).

We have previously reported the use of SPR to characterise the interaction between the immobilized b1 domain of NRP1 (NRP1‐b1) and VEGF‐A‐derived peptides,^[23]^ arginine analogues,^[26]^ and small molecules.[^27^, ^28^] When we attempted to measure the binding affinity of VEGF‐B isoforms to immobilized NRP1‐b1 we observed non‐saturable binding responses. Increasing concentrations of VEGF‐B over immobilised NRP1 led to response units (RU) higher than the theoretical maximum for a 1 : 1 interaction. The shape of the curves remained consistent at low and high concentration and was not typical of sample precipitation (fuzzy heterogeneous curves, commonly observed only at higher concentrations). Observation of the sample plates after analysis also did not show sample precipitation. We consider that the observations are consistent with oligomerization as a form of non‐specific binding. Variation of experimental conditions did not result in a change to this observed behaviour. To circumvent this, we immobilized VEGF‐B_167_ and VEGF‐A_165_ (Figure 2) onto a sensor chip and measured the binding of NRP1‐b1 domain in solution. Using this method, the binding affinity of NRP1‐b1 to immobilized VEGF‐A_165_ was 100‐fold lower than previously observed when the interaction was measured in reverse experimental configuration.^[23]^ Nonetheless, as expected, NRP1‐b1 showed higher affinity for VEGF‐A_165_ (K_D_=9 μM) than VEGF‐B_167_ (K_D_=36 μM) (Figure 2A–D). We then sought to investigate if binding could be blocked by a known inhibitor of the VEGF‐A interaction with NRP1‐b1. EG01377 is a well characterized small molecule that binds to the arginine‐binding site on NRP1‐b1, inhibiting VEGF‐A_165_ binding and subsequent activity.^[28]^ EG01377 shows reproducible binding to NRP1 by SPR.^[28]^ Injection of NRP1‐b1 over immobilised VEGF‐A_167_ and VEGF‐B_167_ in the presence of EG01377 at different concentrations reduced the binding of NRP1‐b1 to both VEGFs (Figure 2E, F). A decrease in the binding response of NRP1‐b1 to VEGF‐B_167_ in the presence of EG01377, similarly to what is observed in the binding to VEGF‐A_165_, suggests that the interaction of VEGF‐B_167_ with NRP1‐b1 also occurs via the arginine binding site of NRP1‐b1 domain and the C‐terminal arginine of VEGF‐B (Figure 1). No binding was observed between EG01377 and VEGFs.

### VEGF‐B and peptides derived from VEGF‐B isoforms bind NRP1

**Figure 3:**
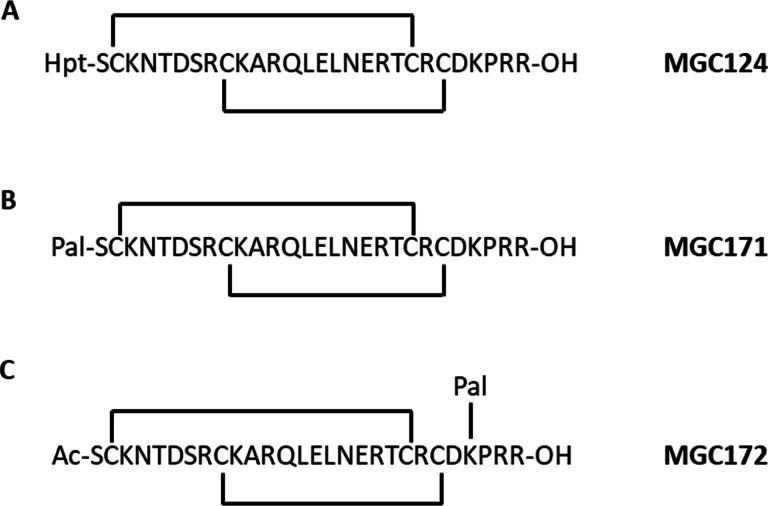
VEGF‐A_165_ derived peptides. Sequences from VEGF‐A_165_ cyclised by disulphide with 6‐heptynoic acid on N‐terminus (A), palmitoyl on N‐terminus (B), and acetyl on N‐terminus, palmitoyl on side chain of lysine (C).

**Figure 4:**
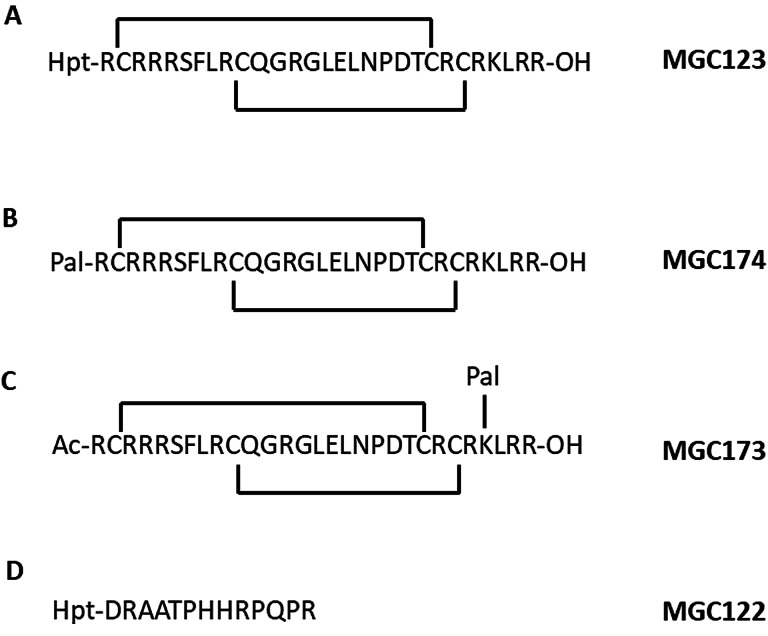
VEGF‐B derived peptides. Sequences from VEGF‐B_167_ cyclised by disulphide with 6‐heptynoic acid on N‐terminus (A), palmitoyl on N‐terminus (B), acetyl on N‐terminus, palmitoyl on side chain of lysine (C). D) Sequence from VEGF‐B_186_ (see also Figure 1).

**Figure 5:**
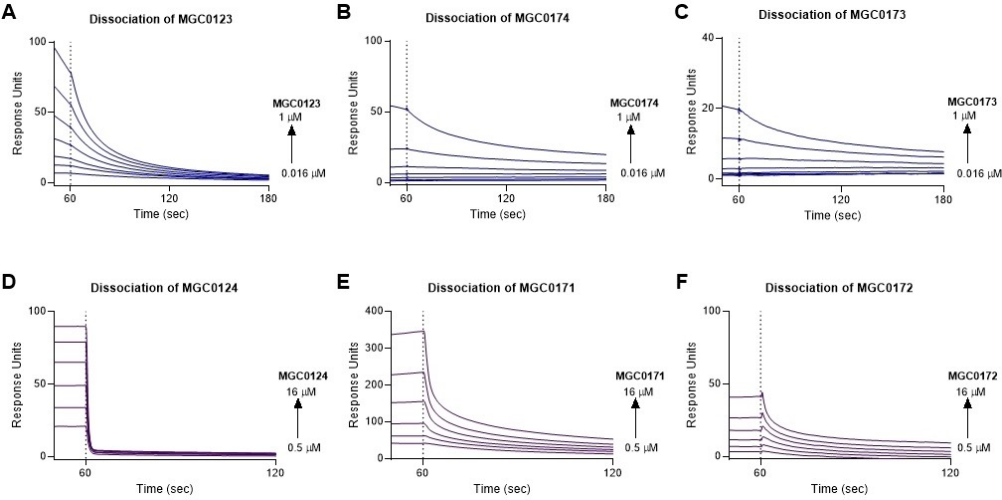
Peptide lipidation slows off‐rates of peptides, SPR sensorgrams show the dissociation phase of peptides after binding to immobilized NRP1‐b1. A) SPR dissociation sensorgram of MGC0123 (0.016–1 μM). B) SPR dissociation sensorgram of MGC0174 (0.016–1 μM). C) SPR dissociation sensorgram of MGC0173 (0.016–1 μM). D) SPR dissociation sensorgram of MGC0124 (0.5–16 μM). E) SPR dissociation sensorgram of MGC0172 (0.05–16 μM). F) SPR dissociation sensorgram of MGC0171 (0.5–16 μM). All peptides were injected over immobilised NRP1‐b1 for 60 sec and the dissociation was measured for either 120 sec or 60 sec. Lipidation state is shown, detailed structures of peptides are in Figures 2 and 3.

**Figure 6:**
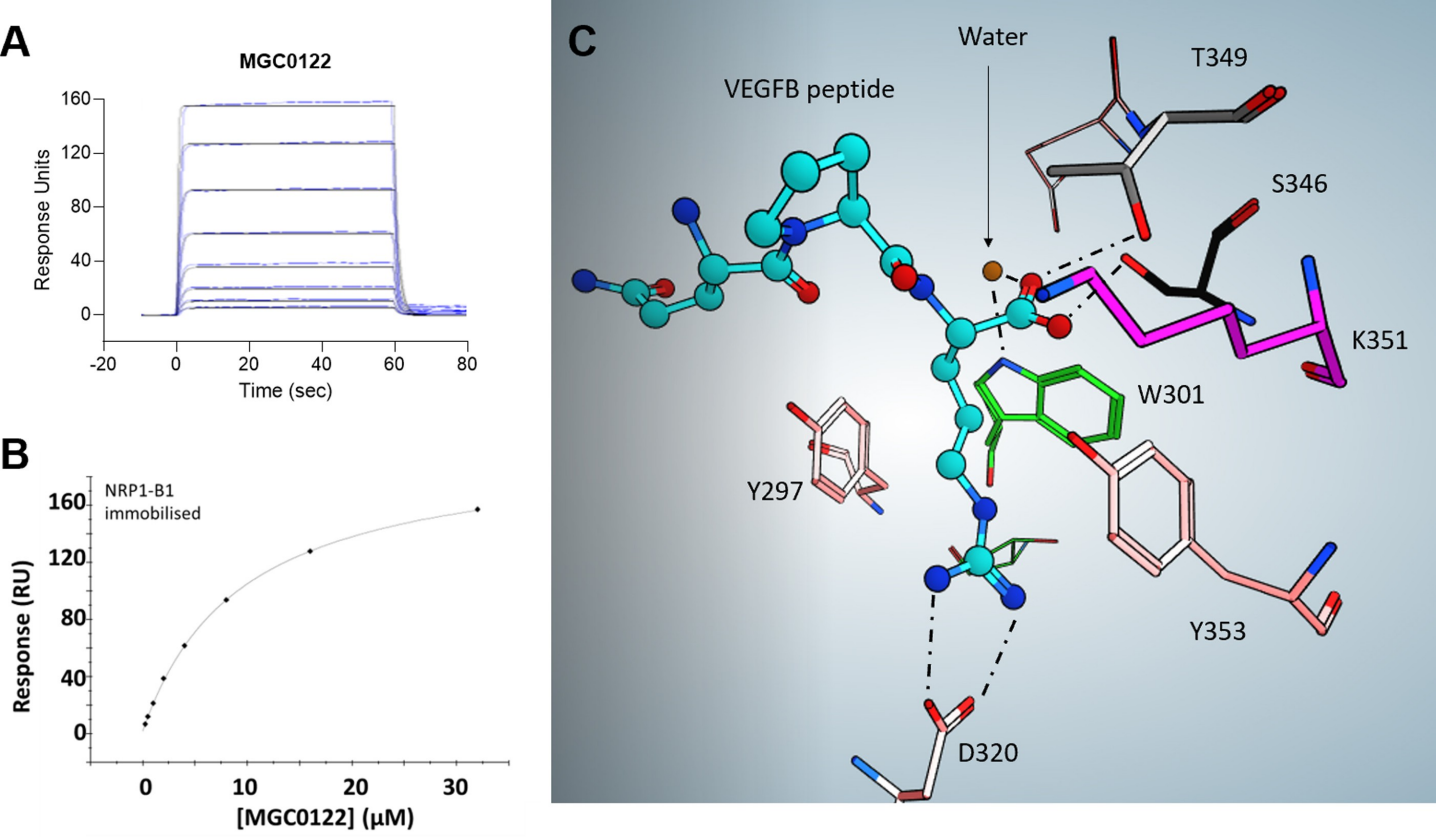
Binding of VEGF‐B derived peptide MGC0122 to NRP1‐b1. A) SPR sensorgram of MGC0122 binding to NRP1‐b1. B) Sensorgram‐derived dose‐response curve of MGC0122 binding to NRP1‐b1. C) X‐ray of MGC0122 bound to the arginine binding pocket of NRP1‐b1 represented as a ball and stick model. The residues making up the binding site are labelled (single amino acid codes). Key H‐bonds are shown as dotted lines.

In order to probe the interaction between NRP1 and VEGF‐B in more detail, we designed several peptides corresponding to the C‐terminal region and compared their binding to NRP1 with VEGF‐B_167_ itself, as well as VEGF‐A ‐ related peptides (Figures 3 and 4). We selected the C‐terminal 29 amino acid sequence of VEGF‐B_167_ and added 6‐heptynoic acid to the N‐terminus (peptide MGC0123) which can be used as a click‐chemistry handle in future assays. For comparison, we also synthesised a VEGF‐A_165_ ‐ derived peptide (MGC0124) closely related to the previously reported EG0086.^[23]^ The effect of palimitoylation at the N‐terminus (peptides MGC0174 and MGC0171) and at the lysines located near the C‐terminus of the VEGF‐B_167_ sequence (R**K**LRR‐OH) and the VEGF‐A_165_ sequence (D**K**PRR) was also determined. The peptides are assumed to be conformationally stabilized through disulphide cysteine bonds as previously observed for the VEGF‐A165 derived peptide EG0086. The VEGF‐B_167_ related peptide MGC0123 (K_D_=0.39±0.09 μM; RT=18.5±4.3 s) showed higher binding affinity and longer residence time to NRP1‐b1 than MGC0124 (K_D_=3.33±0.10 μM; RT=0.7±0 s) (Figure 5A and Table 1). We also synthesized the peptide derived from proteolytically processed VEGF‐B_186_, using the canonical sequence MGC0122 (Figure 1, Figure 4) by analogy with the peptide reported by Makinen.^[21]^ The binding affinity of this peptide was shown to be K_D_=9.55±0.17 μM (Figure 6A, 6B and Table 1).

**Table 1:** SPR binding of VEGF‐A_165_ and VEGF‐B derived peptides to NRP1‐b1.

		SPR binding to NRP1‐b1			Cell‐based activity (HUVECs)
Peptide	VEGF isoform	K_D_ [μM]	Residence time [s]^[a]^	Receptor occupancy [%]	IC_50_ [μM]
MGC0123	VEGF‐B_167_	0.39±0.09	18.5±4.3	54±3	ND
MGC0124	VEGF‐A_165_	3.33±0.10	0.7±0.0	84±1	ND
MGC0174	VEGF‐B_167_	0.33±0.05	14.9±21; 267.9±21.9	47±3	0.3
MGC0171	VEGF‐A_165_	3.32±0.16	5.3±0.5; 95.0±28.2	415±17	1.7
MGC0173	VEGF‐B_167_	0.13±0.03	4.6±2.6; 161.6±48.2	10±1	2.0
MGC0172	VEGF‐A_165_	1.22±0.25	7.4±1.3; 119.3±10.6	22±1	10.4
MGC0122	VEGF‐B_186_	9.55±0.17	2.17±3.01	ND	ND

[a] Second residence time calculated from two‐state model. ND=Not determined.

### X‐ray analysis of the VEGF‐B derived peptides binding mode

In order to unequivocally establish the binding mode of the VEGF‐B derived peptides we undertook X‐ray crystallography studies. Crystallisation trials were set up for various VEGF‐B‐derived peptide/neuropilin complexes and MGC0122 (Hpt‐DRAATPHHRPQPR), which was the shortest peptide of those reported here, was successfully crystallised with NRP1‐b1 domain. The complex exhibited symmetry, with 2 NRP1‐b1 molecules per asymmetric unit. The structure of the NRP1‐b1 complexed with MGC0122 reveals the presence of the ligand in both NRP1‐b1 molecules. The binding site is composed of amino acids Y297, Y353, W301, T349, S346 and D320 (Figure 6C, K351 is also visible in the foreground) previously identified as the binding place for the C‐terminal domain of VEGF‐A_165_.^[29]^ The peptide's carboxylate group forms direct H‐bonds with residues T349, S346 and Y353 of the NRP1‐b1 domain. In addition, a water molecule mediates H‐bond between the carboxylate group and NE1 of W301 and OE1 of E348. The OD1 and OD2 atoms of D320 form H‐bonds with the N‐ atom of the guanidine part of ligand. In both NRP1‐b1‐MGC0122 complexes within the asymmetric unit only the C‐terminal part of the peptide with its QPR amino acids sequence is ordered and identifiable in the electron density maps while the remainder of the peptide is highly disordered. Even though the crystal packing provides sufficient space to accommodate the peptide and there are areas of unaccounted but disconnected electron density the full ligand could not be modelled with confidence. This structural information contributes to the overall characterisation together with SPR and functional data. Both VEGF‐B_167_ and VEGF‐B_186_ derived peptides can be considered mimics of VEGF‐B, and the comprehensive picture suggests that together with VEGF‐A_165_ they share a common ligand‐binding site on neuropilin‐1. Although they all interact with the b1 domain of neuropilin, in a C‐terminal arginine‐dependent fashion, their apparent affinities of interaction differ. The poor affinity ligand, (VEGF‐B_186_‐related), was able to crystallise most likely as it was smaller and more amenable to crystal packing. Numerous attempts to crystallise the larger cyclic peptides were not successful.

### VEGF‐A ‐stimulated phosphorylation of ERK in endothelial cells is potently blocked by a VEGF‐B derived peptide

**Figure 7:**
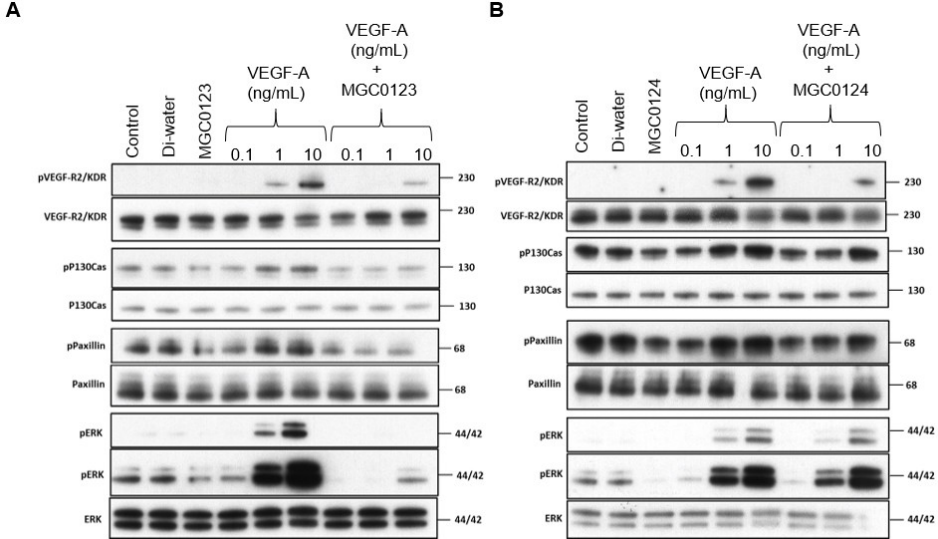
Effect of VEGF ‐ derived peptides on VEGF‐A ‐stimulated phosphorylation of downstream signalling proteins as probed by Western blot. A) VEGF‐B_167_ derived peptide MGC0123. B) VEGF‐A_165_ derived peptide MGC0124. HUVECs were pre‐treated for 30 min with peptide (10 μM) then stimulated for 10 minutes with VEGF‐A_165_.

VEGF‐B has been shown to compete with VEGF‐A for binding to NRP1,^[27]^ so we decided to investigate further if treatment with VEGF‐B peptides MGC0123 and MGC0124 were antagonistic to VEGF‐A downstream signalling in human endothelial cells. The peptides were developed around the amino acid residues on VEGF‐B which have been shown to bind to NRP1. These are different from the region on VEGF‐B, which bind to VEGFR2. Furthermore, we have previously shown that peptides designed around the VEGF‐A determinants for NRP1 binding have high specificity and are unable to bind VEGFR2.^[30]^ The effect of peptides MGC0123 (VEGF‐B_167_ derived) and MGC0124 (VEGF‐A_165_ derived) on VEGF‐A canonical signalling pathways in endothelial cells was probed by Western blot analysis. VEGF‐A_165_ dose dependent increases in VEGFR2 and ERK phosphorylation were dramatically inhibited in cells treated with 10 μM MGC0123. VEGF‐A mediated increases in P130Cas and Paxillin phosphorylation were also inhibited (Figure 7A). In contrast, treatment with the VEGF‐A_165_ derived peptide, MGC0124, although strongly reducing VEGFR2 phosphorylation, had little effect on the other signalling proteins (Figure 7B).

### Lipidation of the peptides and their binding affinity for NRP1

**Figure 8:**
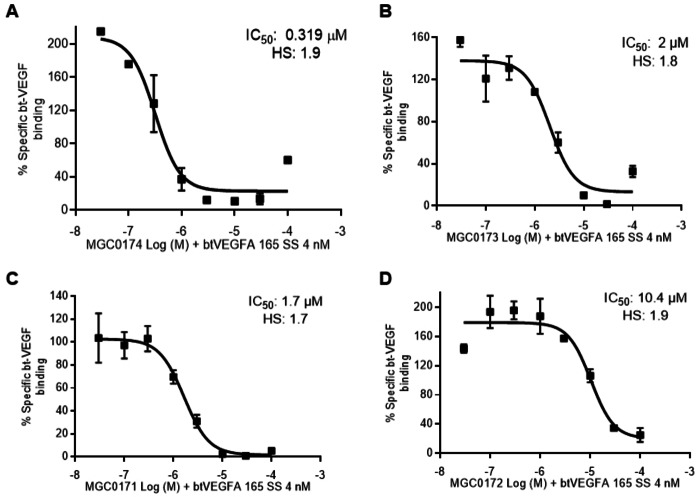
Inhibition of bt‐VEGF‐A_165_ binding to NRP1 in HUVEC cells by lipidated peptides. A) N‐terminal palmitoyl VEGF‐B_167_ derived peptide MGC0174. B) Lysine palmitoyl peptide MGC0173. C) N‐terminal palmitoyl VEGF‐A_165_ derived peptide MGC0171. D) Lysine VEGF‐A_165_ palmitoyl peptide MGC0172. Interaction of peptides with NRP1 was assessed by their inhibition of bt‐VEGF‐A_165_ (4 nM) binding to NRP1 in HUVEC cells using a streptavidin‐ horseradish peroxidase detection.

We then designed four additional peptides with added lipid chains (Figures 3 and 4). We investigated the effect of lipidation on the N‐terminus (MGC0171 and MGC0174) and on the side chain of a lysine in close proximity to the C‐terminus (MGC0172 and MGC0173) as shown (Figure 5). For the SPR analysis of the lipidated peptides we fitted data to a two‐state reaction model to account for the nonspecific binding of the palmitoyl side chains. Since the model assumes two binding events it provides two association (*k*_a_) and dissociation (*k*_d_) rates, and the overall equilibrium dissociation constant is calculated using the equation: K_D_=K_D1_(1+K_D2_), in which K_D1_ and K_D2_ are obtained as K_D_=*K*_a_/k_d_. The inverse of the dissociation rate corresponds to the residence time. A notable effect of lipidation on the dissociation phase of peptides from NRP1 was noted (Figure 5). VEGF‐B peptides (Figure 5 A,B,C) showed very slow off rates on lipidation but even the rapid dissociation of VEGF‐A peptides was slowed (Figure 5 D,E,F). Lipidation increased the residence time of the peptides while maintaining overall affinity (Table 1). However, MGC0173 (10±1 % occupancy) and MGC0172 (22±1 % occupancy) showed a decrease in the occupancy of the receptor, suggesting lipidation in this position may add steric hindrance to the binding site. Conversely, MGC0171 (415±17 % occupancy) showed non‐stoichiometric binding to NRP1‐b1, suggesting possible aggregation or non‐specific binding (Table 1). The lipidated peptides were also tested for inhibition of VEGF‐A_165_ binding to NRP1 in a cell‐based assay (Figure 8). N‐terminal palmitoyl groups did not adversely affect binding (Figure 8A, 8 C) whereas Lysine side chain palmitoylation (Figure 8B, 8D) resulted in decreased potency, consistent with reduced receptor occupancy. MGC0174 emerged as a lead compound (K_D_=0.33±0.05 μM; RT=14.9±21 and 267.9±21.9 s; 47±3 % occupancy; IC_50_=0.3 μM).

### Stability of the lipidated peptides in plasma

**Figure 9:**
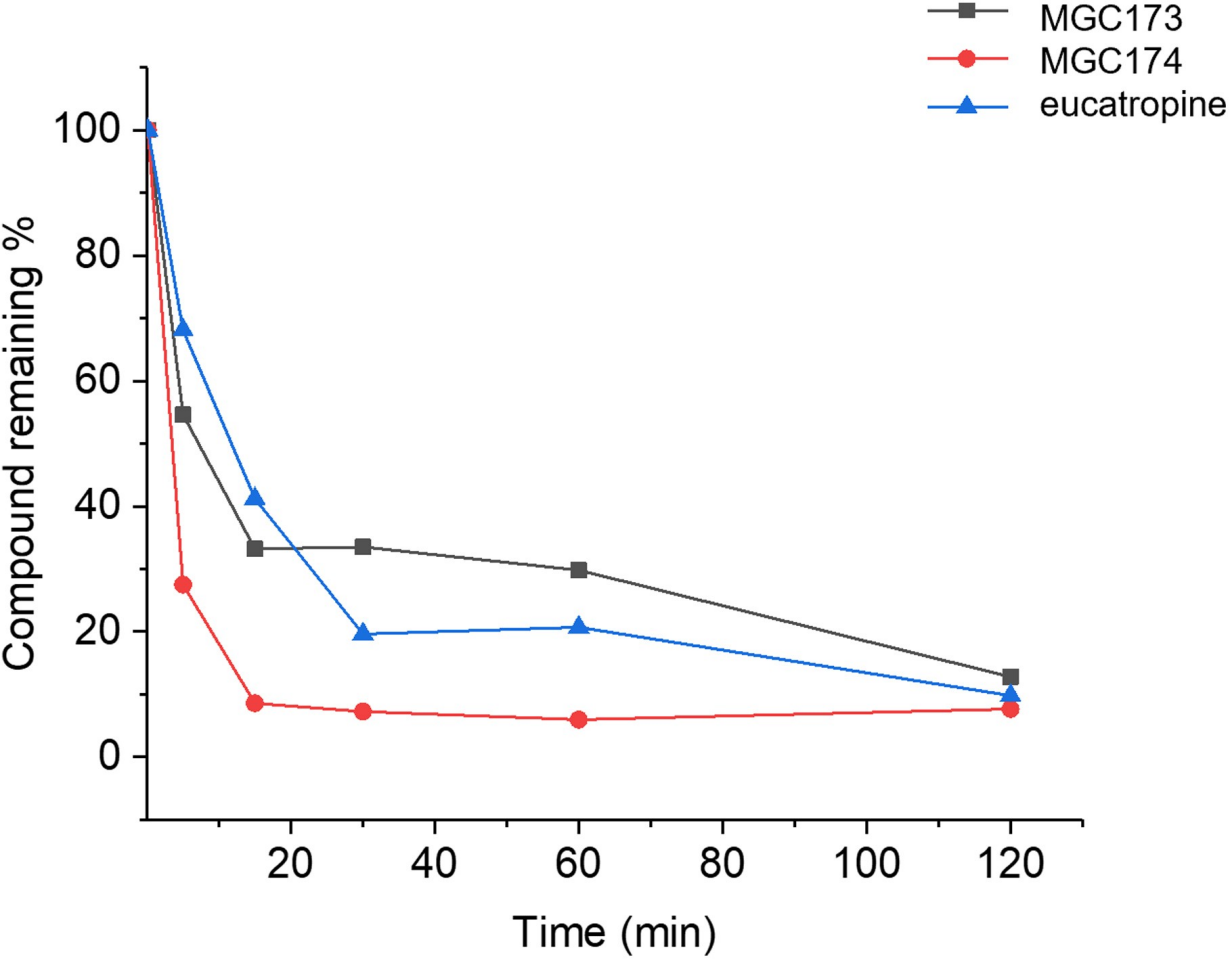
Stability of VEGF‐B_167_ lipidated peptides in mouse plasma determined by MSMS analysis. The stability of the peptides is shown relative to the control eucatropine. Samples were analysed in triplicate (n=1).

Peptides derived from the C‐terminus of VEGF‐A are unstable in plasma with EG0086 showing complete degradation after 5 min with proteolytic cleavage of the C‐terminal arginine.^[23]^ The VEGF‐B_167_ derived peptides with palmitoyl fatty acid groups at the N‐terminus, MGC0174, and at the lysine side chain of the C‐terminal sequence LKRR, MGC0173, were assessed for stability in mouse plasma using liquid chromatography‐mass spectrometry. The data (Figure 9) showed that MGC0174 quickly degraded to low levels after 15 min, whereas MGC0173 maintained much better levels throughout the course of the experiment and gave a half‐life of 51 min. The positive control for this assay was eucatropine which degraded within literature parameters.^[31]^ This indicates that side chain palmitoylation close to the C‐terminal cleavage site imparts resistance to cellular proteases such as plasmin.

## Discussion

In summary, we used SPR to characterize the binding of VEGF‐B_167_ to NRP1‐b1 and showed that binding can be inhibited by the potent NRP1 inhibitor EG01377. EG01377 binds NRP1‐b1 at its well‐defined CendR arginine binding site. The C‐terminal region of VEGF‐B_167_ has close homology to VEGF‐A_165_, including the C‐terminal arginine residue. We synthesised novel peptides derived from the C‐terminal residues of VEGF‐A_165_ and VEGF‐B_167_ which show potent binding to NRP1‐b1. The striking inhibitory effects of the VEGF‐B peptides on VEGF‐A‐induced ERK activation and VEGFR2 tyrosine phosphorylation indicate a major role for NRP1 in mediating VEGF signalling in endothelial cells via NRP1, most likely as a result of heterodimerisation of NRP1 with VEGFR2.^[32]^ This conclusion is consistent with our findings that VEGF‐B‐derived peptides bind to the same well‐defined binding site in the NRP1 b1 domain as VEGF‐A, and therefore act by inhibiting VEGF‐A binding to NRP1 in endothelial cells, thus reducing VEGF‐A stimulation of ERK activation and VEGFR2 tyrosine phosphorylation. Surprisingly, VEGF‐B_167_ derived peptides are more potent than their VEGF‐A homologs in both binding and functional assays. The relatively potent effect of the VEGF‐B_167_ derived peptide MGC0123 on ERK phosphorylation is intriguing and poses the question if this is downstream of VEGFR1 or VEGFR2 phosphorylation. ERK activation in human endothelial cells (HUVECs), is thought to be predominantly a result of VEGFR2 tyrosine kinase activation by VEGF‐A_165_, though VEGF‐B is also known to activate ERK in other cell types such as dopaminergic neurons.^[33]^ Therefore, we cannot rule out a more specific role for the VEGF‐B_167_ peptide in blocking VEGF‐A mediated VEGFR1 induced ERK phosphorylation. The simplest explanation though, is that the slow off‐rates observed in the VEGF‐B_167_ derived peptide NRP1 binding analyses are responsible for an increased functional potency in blocking VEGF‐A mediated ERK phosphorylation. HUVECs express both VEGFR1 and VEGFR2 and VEGF‐A_165_ binds both receptors. VEGF receptors may also form heterodimers further complicating the picture.^[32]^ Therefore, we cannot rule out a more specific role for the VEGF‐B_167_ peptide in blocking VEGF‐A mediated VEGFR1 induced ERK phosphorylation.

Lipidation increases residence time and plasma stability of all peptides in this study. The lipidated VEGF‐B_167_ derived peptides MGC0173 and MGC0174 showed micromolar inhibition of VEGF‐A in HUVEC cells and could be useful tools for VEGF‐B related research. In particular the recent hypothesis for VEGF‐B as an insulin resistance factor in type II diabetes^[16]^ and its similar importance in NAFLD^[18]^ would indicate the potential for blockade of the VEGF‐B function. Lipidated peptides are mainstays of type II diabetes treatment and provided some stabilization as indicated in this study.^[25]^ While mimicking the VEGFR1 binding site could activate the neuroprotective functions of VEGF‐B^[15]^ the peptides described here could be specific blockers of VEGF‐B function.

## Experimental Section

**Materials**: All surface plasmon resonance reagents, chips and buffers were from GE‐Healthcare, Little Chalfont, UK (now Cytvia). Peptides were purchased from Peptide Protein Research, Fareham, UK. bt‐VEGF165 50 μg/mL (1200 nM) was from AcroBiosystems, Newark, USA. EGM Endothelial Cell Growth Medium (Lonza, USA). Streptavidin‐horseradish peroxidase DY998 (R&D Systems). Other materials are indicated in the detailed methods.

**Sequence alignments**: Protein sequences were obtained from UniProtKB access numbers P14692‐4 (VEGF‐A_165_, human) and P49765‐2 (VEGF‐B_167_, human). Sequence alignments were performed using Clustal Omega (The European Bioinformatics Institute).

**Peptide synthesis**: Peptides were designed in ‐house and synthesised by PeptideSynthetics (Peptide Protein Research Ltd) to >95 % purity determined by HPLC.

**Surface plasmon resonance**: SPR measurements were performed using either Biacore T200 or Biacore 4000 at a constant temperature of 25 °C. All sensor chips, stock buffers, and immobilisation reagents were purchased from GE Healthcare/Cytvia and were prepared according to the manufacturer's instructions.

*Chip preparation*: PBS containing 0.05 % surfactant P20 was used as the running buffer during immobilisation. Proteins were immobilised onto a CM5 chip using random amine coupling following a standard procedure using the conditions specified in Table 2. A flow cell upstream of the treated flow cell was used as a reference which allowed removal of non‐specific binding to the chip.

**Table 2:** Protein immobilisation conditions.

Protein	Concentration	pH	Immobilisation level
NRP1‐b1	20 μg/mL	5	1178
VEGF‐A_165_	20 μg/mL	4.5	2151
VEGF‐B_167_	40 μg/mL	4.5	2735

*Binding of NRP1‐b1 to VEGFs*: Dose responses were obtained by injecting NRP1‐b1 in PBS over the immobilized VEGFs for 60 s, using two‐fold dilutions from 64 to 0.25 μM. Steady‐state binding affinities were calculated assuming a 1 : 1 interaction.

*EG01377 competition assay*: NRP1‐b1 (5 μM) was injected over immobilized VEGF‐A_165_ and VEGF‐B_167_ with increasing concentrations of EG01377 for 60 s. For inhibition of NRP1‐b1 binding to VEFG‐B_167_, EG01377 was injected at concentrations ranging from 6.25 to 50 μM and for inhibition of binding to VEGF‐A_165_ concentrations ranging from 1.25 to 10 μM were used.

*Kinetic measurements of peptides*: Peptides were injected over immobilized NRP1‐b1 for 60 s, followed by 120 or 180 s dissociation. The concentration ranges are shown in Table 3. The surface was regenerated with 1 M NaCl between injections. The SPR response curves were fit to a binding model to obtain association (*k_a_*) and dissociation (*k_d_*) rates which were then converted into equilibrium dissociation constant (*K_D_*) and residence time. For a 1 : 1 binding model K_D_=*k_a_*/*k_d_* and for a two‐state binding model K_D_=K_1_(1+K_2_). Residence time was calculated as 1/*k_d_*.

**Table 3:** Details of peptides used for SPR kinetic analysis.

Peptide	Molecular weight	Concentration range [μM]	Binding model
MGC0122	1697	32 to 0.50	1 : 1
MGC0123	3710	4 to 7.8E‐3	1 : 1
MGC0124	3472	64 to 0.25	1 : 1
MGC0171	3602	16 to 0.25	two‐state
MGC0172	3644	16 to 0.25	two‐state
MGC0173	3882	1 to 7.8E‐3	two‐state
MGC0174	3840	1 to 7.8E‐3	two‐state

**Protein purification**: Frozen cell pellets from 2 L *E.coli* Rosetta (DE3) were resuspended in lysis buffer, 20 mM Tris (pH 7.9), 20 mM imidazole, 250 mM NaCl, supplemented with one tablet of protease inhibitors (Roche) and lysed by sonication. Soluble proteins were separated by centrifugation, and loaded onto a pre‐equilibrated 5 mL HisTrap HP column (GE Healthcare) connected to an ÅKTA purifier. NRP1‐b1 domain was eluted using an imidazole gradient over 10 column volumes, with the final buffer being 20 mM Tris (pH 7.9), 600 mM imidazole, 250 mM NaCl. After removal of a poly‐histidine tag by TEV protease cleavage NRP1‐b1 protein was further purified using a Superdex 75 16/60 gel filtration column equilibrated with 25 mM MES (pH 6.0), 40 mM NaCl. Final purification step included anion exchange chromatography with SP FF cation exchange column (GE Healthcare) using a salt gradient over 30 column volumes, final buffer being 25 mM MES (pH 6.0), 500 mM NaCl. Pure NRP1‐b1 protein was dialysed against 20 mM Tris‐HCl (pH 7.9), 50 mM NaCl at 4 °C overnight.

### X‐ray crystal structure acquisition

*Crystallization*: NRP1‐b1 was concentrated to 10 mg/mL (0.5 mM), as determined by the absorbance at 280 nm using a Nanodrop spectrophotometer (N100). 10 μL of protein were mixed with 1 μL of concentrated solution of the peptide and incubated for 1 h at 4 °C. Concentrations of peptide stock solutions vary according to peptide solubility and are usually between 10–50 mM in 100 % DMSO. Protein can tolerate maximum 10 % DMSO. Hanging drops were setup by mixing 1 μL of protein‐peptide mixture with 1 μL reservoir solution and 0.35–0.5 μL of seeds of apo‐NRP1‐b1 at 1/10 dilution. Reservoir conditions between 10 to 30 % PEG 3350+0.2 M ammonium chloride, in increasing steps of 2 % were screened.

*Seed preparation*: A whole drop with crystals of apo‐NRP1‐b1 was picked up and 50 μL of reservoir solution was added to it together with one bead from the Hampton research‘s seeding kit. Mixture was vortexed for 90 s. 5 μL of this suspension was diluted with 45 μL of reservoir solution to prepare 1/10 dilution used for seeding.

*Data collection*: Crystal was transferred in cryoprotectant solution consisting of 20 % Ethylene Glycol+5 mM peptide in 12 % PEG 3350+0.2 M ammonium chloride and flash frozen by plunging directly into liquid nitrogen. Data sets were collected at Diamond LS, I04 beamline.

*Data processing, structure solution, model refinement and building*: Data were processed using the xia2‐3dii automated routine. Initial phases were calculated using the PHASER programme of CCP4 crystallographic software suite.^[34]^ Coordinates of human NRP1‐b1 domain (PDB ID=1KEX) were used as search model. The molecular replacement solution model was refined by REFMAC5 refinement programme of CCP4. After 10 cycles of refinement with TLS parameters, 2Fo−Fc and Fo−Fc difference maps were calculated and protein structure was examined. After fitting all protein residues and new cycle of refinement, Fo−Fc difference maps were calculated and used for searching for density corresponding to the peptidic ligand, using COOT.^[35]^ Ligand was built by modelling the amino acids of the peptide, starting from C‐terminal Arg into the identified density. After ligand fitting and several cycles of refinement with REFMAC5, water molecules were added using COOT. Table 4 includes data and refinement statistics; Figure 6 shows the final model. Validation of final model‘s geometry was performed by using the validation tools of COOT. Final coordinates and the structure factors were deposited to PDB with PDB ID: 7P5 U.

**Table 4:** Data collection and refinement statistics.

Data collection	
X‐ray source	Diamond I04
Space group	P 21 21 21
Cell constants a, b, c, *α*, *β*, *γ*	48.50 Å, 74.35 Å, 91.43 Å, 90.00°, 90.00°, 90.00°
Resolution (Å)	57.68–1.60 (1.67–1.60)
% Data completeness	99.1 (94.3)
R_*merge*_	0.065 (0.67)
CC_1/2_	0.999 (0.734)
*<I/σ*(*I*)*>*	14.5 (2.4)
Wilson B‐factor (Å^2^)	17.0
Values in brackets refer to the highest resolution shell	

Refinement	
Refinement program	REFMAC 5.8.0267
R_*work*_, R_*free*_	0.188, 0.214
R_*free*_	5 %
Average B, all atoms (Å^2^)	20.0
RMSD bond lengths (Å)	0.015
RMSD bond angles (°)	1.944
Ramachandran outliers %	0

**Cell culture**: Human umbilical vein endothelial cells (HUVECs) were cultured in t75 cm2 flasks for a maximum of 2 passages in EGM Endothelial Cell Growth Medium (#CC‐3124, Lonza, USA) supplemented with 10 % FBS. Cells were split into 6‐well‐plates and allowed to grow to ∼80 % confluence. Cells were starved by incubating in 0.5 % FBS serum EGM overnight and serum‐free EGM for 1 h directly before stimulation. Human DU145 cells for the cell‐based assay (see below) were cultured in t75 cm2 flasks in DMEM supplemented with 10 % FBS.

**Immunoblotting**: Cells were pre‐treated with the indicated peptide (10 μM) for 30 minutes prior to treatment with VEGF‐A_165_ at the indicated concentrations for 10 minutes. Cells were lysed in a solution containing Tris⋅HCl (pH 7.5, 50 mm), Triton X‐100 (1 %), NaCl (150 mm), EDTA (5 mm), complete protease inhibitor (Roche) and phosphatase inhibitors I and II (Sigma) and analysed by SDS‐PAGE with 4–12 % Bis⋅Tris gels (Nupage, Invitrogen), followed by electrotransfer onto Invitrolon PVDF membranes (Invitrogen). Membranes were blocked with non‐fat dry milk (5 % w/v) and Tween‐20 (0.1 % v/v) in tris‐buffered saline (TBS‐T), for 1 h at room temperature, before being probed with the primary antibody by overnight incubation at 4 degrees C, followed by incubation for 1 h at room temperature with a horseradish‐ peroxidase‐linked secondary antibody (Santa Cruz) and detection with the aid of ECL plus reagents (GE Healthcare, Little Chalfont, UK), by the manufacturer's protocol.

**Cell based binding assay for bt‐VEGF binding to NRP1‐b1**: A flat‐bottomed 96 well plate was coated with 100 μL 10 μg/mL Poly‐D‐lysine (110 μL in 10890 μL PBS) and incubated for 1 hour at R.T. DU145 cells were seeded at a concentration of 20,000 DU145 cells/well in DMEM with 10 % FBS and incubated for 4 hours. Cells were then infected with 0.3 μL per well of adenovirus expressing NRP1 (stock at 1×1012 vp/mL) and incubated for 40 hours. The media was removed followed by washing cells twice in 200 μL of PBS per well. The plate was placed on ice and 50 μL of binding medium was added per well for total binding. Next, 50 μL of 100‐fold excess (100×btVEGF‐A_165_) of unlabelled‐VEGF (diluted in binding medium) was added per well for non‐specific binding. Peptides (50 μL) at the indicated concentrations were diluted in binding medium and added to specified wells. bt‐VEGF (50 μL of 4 nM; diluted in binding medium) was added per well for both total and non‐specific binding and samples. The plate was incubated at 4 °C for 2 hours. The plate was then placed on ice and the medium was removed followed by gently washing the cells 3 times in 200 μL of PBS per well.

Streptavidin‐horseradish peroxidase (100 μL; dilute to the working concentration specified on the vial label using PBS+1 % BSA=1 : 200) was added to each well. The plate was sealed and incubated for 30 minutes at room temperature. Three more washes with 200 μL PBS were done. Then, 100 μL of substrate solution was added to each well (protected from light). Colour Reagent A and B were mixed together in equal volumes within 15 minutes of use. The plate was sealed and incubated for 20 minutes at room temperature. Stop solution (50 μL) was added to each well ensuring even mixing. The plate was read using Tecan Genios plate reader using a 450 nm cut‐off filter and also with a reference 595 nm cut‐off filter. Nonspecific binding was determined in the absence of NP‐1 coated on the microplate.

**Peptide stability in mouse plasma**: Mouse plasma stability tests were performed by Cyprotex. In brief, peptides were incubated with mouse plasma (adjusted for pH 7.4, 37 °C) to a final concentration of 10 μM and incubation volume of 500 μL (0.24 % DMSO). After the incubation period (0, 5, 15, 30, 60, and 120 min), the reactions were stopped by transferring 50 μL of incubate to 150 μL acetonitrile containing internal standard. Plasma proteins were precipitated out by centrifuging at 3,000 rpm for 45 min at 4 °C. The supernatant was diluted 1 : 1 with water and analysed by LC‐MS/MS.

**Data availability**: Data available on request from the authors The data that support the findings of this study are available from the corresponding author upon reasonable request. Some data may not be made available because of privacy or ethical restrictions.

## Conflict of interest

The authors declare no conflict of interest.
